# Transesophageal echocardiography measurements of aortic annulus diameter using biplane mode in patients undergoing transcatheter aortic valve implantation

**DOI:** 10.1186/1476-7120-11-5

**Published:** 2013-01-30

**Authors:** Kambiz Shahgaldi, Cristina da Silva, Magnus Bäck, Andreas Rück, Aristomenis Manouras, Anders Sahlén

**Affiliations:** 1 Karolinska Institutet, Department of Cardiology, Karolinska University Hospital, Huddinge, 141 86, Stockholm, Sweden; 2 School of Technology and Health, Royal Institute of Technology, Huddinge, 141 52, Sweden

**Keywords:** Transcatheter aortic valve implantation, Transesophageal echocardiography, Aortic annulus, Biplane-mode, Three-dimensional transesophageal echocardiography

## Abstract

**Background:**

Aortic stenosis (AS) is a relevant common valve disorder. Severe AS and symptoms and/or left ventricular dysfunction (EF <50%) have the indication for aortic valve replacement (AVR). Majority of the patients with AS are elderly often with co-morbidities and generally have high preoperative risk. Transcatheter aortic valve implantation (TAVI) is offered in this group. Four different sizes of Corevalve prosthesis are available. Correct measurement of aortic size prior to TAVI is of great important to choose the right prosthesis size to avoid among others paravalvular leak or prosthesis patient mismatch.

Aim of the study is to assess the aortic annulus diameter in patients undergoing TAVI by biplane (BP) mode using transesophageal echocardiography (TEE) and compare it to two-dimensional (2D) transthoracic echocardiography (TTE) and 2DTEE using three-dimensional (3D) TEE as reference method.

**Methods:**

The study population consisted of 50 patients retrospectively (24 men and 26 women, mean age 85±8 years of age) who all had undergone echocardiography examination prior to TAVI.

**Results:**

The mean aortic annulus diameter was 20.4±2.2 mm with TTE, 22.3±2.5 mm with 2DTEE, 22.9±1.9 mm with BP-mode and 23.1±1.9 mm with 3DTEE. TTE underestimated the mean aortic annulus diameter in comparison to transesophageal imaging modalities (p<0.001). Using 3DTEE, 2% of patients were unsuitable for TAVI due to a too-small AoA (n=1). This figure was similar with BP (4%, n=2; p=1.00) but considerably larger with 2DTTE (36%, n=18; p < 0.001) and 2DTEE (12%, n=6; p=0.06). There was a strong correlation between BP-mode and 3DTEE for assessment of aortic annulus diameter (r-value 0.88) with small mean difference (−0.2±0.9 mm) whereas the other modalities showed larger 95% confidence interval and modest correlation (2DTTE vs. 3DTEE, –6.3 to 0.9 mm, r=0.64 and 2DTEE vs. 3DTEE, –4.8 to 3.2 mm, r=0.61).

**Conclusion:**

A multi-dimensional method is preferred to assess aortic annulus diameter in TAVI patients since there is risk of underestimation using single plane. Biplane mode is the method of choice in view of speedy post-processing with no need for expensive dedicated software. Lastly, single plane methods lead to misclassification of patients as unsuitable for TAVI. This may be of major clinical importance.

## Background

Aortic stenosis (AS) affects nearly 5% of patients >75 years of age [1,2]. Aortic valve replacement (AVR) is indicated in severe AS with symptoms or left ventricular (LV) dysfunction, as the prognosis is otherwise poor [3]. Transcatheter aortic valve implantation (TAVI) is a relatively new procedure which is increasingly being offered in elderly or high-risk patients with AS that are considered unsuitable for open heart surgery due to co morbidities [4-6]. Accurate pre-operative evaluation of aortic annulus (AoA) diameter is important as it determines the selection of prosthesis size. Implantation of an appropriately-sized prosthesis enables the procedure to be performed with a smaller risk of serious complications including aortic root damage, AV-block, pro**s**thesis embolization or paravalvular regurgitation [7]. The most common modalities for measuring AoA are transthoracic echocardiography (TTE), transesophageal echocardiography (TEE) and coronary angiography. However, as these are two-dimensional (2D) methods, the AoA is measured in only one image plane. This introduces a risk that the maximal diameter is underestimated, with obvious and potentially serious clinical consequences as illustrated by a post-TAVI incidence of severe PPM of approximately 2-6% [8,9].

To date, there is no established gold standard technique for measuring AoA prior to TAVI. Recent studies have compared TTE, 2DTEE, three-dimensional (3D) TEE, multislice-computed tomography (MSCT) and magnetic resonance imaging (MRI) for the determination of AoA in this group [10-12]. MSCT has been suggested for improved pre-procedural annular measurement and prosthesis sizing [13-16]. 3DTEE has been demonstrated to measure AoA size accurately when compared to dual-source CT and intra-operative measurements, as it allows the maximal diameter of the annulus to be directly visualized in short axis view after careful alignment [12,17]. A recent study demonstrated that AoA measured by 3DTEE is larger than with 2DTEE and concluded that this had considerable impact on choice of prosthesis size [18].

2DTTE is the first method of choice according to the recent European Association of Echocardiography and American Society of Echocardiography (EAE/ASE) recommendations for the use of echocardiography in transcatheter intervention [19]. If measurements are close to critical cut-offs for valve size selection, or if there is difficulty in measuring annulus size due to calcification, the authors suggest that TEE and/or 3DTEE may be necessary. While 3DTEE has emerged as a promising technique, this technique enables the annulus to be measured during post-processing at a dedicated work station. On the contrary, biplane (BP) mode is an attractive complement to 2D imaging as it enables multi-planar imaging to be performed, and AoA measurements taken, on-line. In BP-mode, two simultaneous views of the annulus are recorded simultaneous. As previous research in this field has not formally shown the validity of BP-mode in this setting, and as prosthesis sizing is essential in TAVI patients, we wished to investigate this important methodological question.

We tested the hypothesis that measurement of AoA dimension using BP-mode in patients with severe AS referred for TAVI results in more accurate and reproducible measurements compared to 2D techniques, using 3DTEE as reference method.

## Material and methods

### Patient population

Fifty patients were studied retrospectively with severe AS referred to our centre for TAVI (Medtronic-CoreValve Inc., Minneapolis, MN) and who had undergone 2DTTE, 2DTEE, BP-mode and 3DTEE acquisition, were included in this retrospective study. All examinations were clinically indicated as pre-TAVI work-up. The study complied with the declaration of Helsinki.

### 2D transthoracic echocardiography

**Figure 1:**
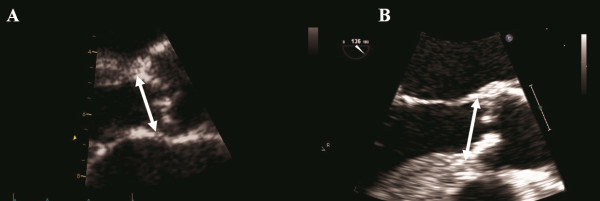
**A and B. Aortic annulus measurements performed by transthoracic and transesophageal echocardiography.** Aortic annulus dimension is indicated by the arrow.

All patients underwent complete echocardiography examination in the left lateral decubitus position using commercially available ultrasound system (iE33, Philips, Andover, Massachusetts, USA) with a 1–5 MHz transducer (S5-1). Analyses were performed at a dedicated workstation using a commercially available software package (EchoPAC, GE, Horten, Norway). The severity of AS was assessed in agreement with EAE/ASE recommendations [20] and the degree of aortic regurgitation was assessed according to current guidelines [21]. All measurements of AoA were taken during systole and recorded as an average from 3 beats. As shown in Figure 1A, AoA was recorded as the inner edge to inner edge distance measured from the junction of the aortic right coronary cups with the septal endocardium to the junction of the non-coronary cups with the mitral valve posteriorly. Annular calcification was included [15,20,22]. During TTE, AoA was measured in zoom mode in the parasternal long-axis view. All measurements were performed blinded from each other by two experienced investigators.

### 2D and 3D transesophageal echocardiography

The Philips iE33 system (Philips, Andover, Massachusetts, USA) was used with a TEE (X7-2t) transducer allowing both 2D and 3DTEE images. This matrix array transducer provides high-resolution real-time 3D imaging. AoA was acquired in mid esophageal position using an image plane between 120° – 150°.

During 2DTEE image acquisition, every effort was made to ensure that the largest annulus diameter was obtained using zoom mode. Measurements were performed in the same manner as with 2DTTE (Figure 1B).

**Figure 2:**
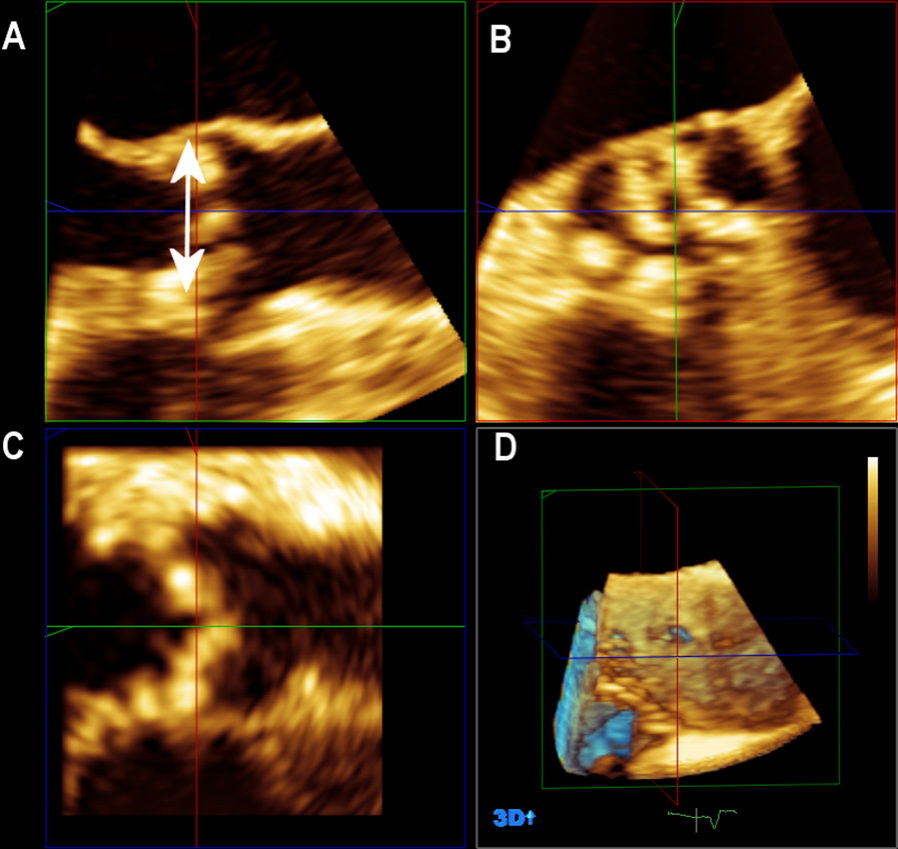
**Reconstruction of the three-dimensional transesophageal echocardiography in 3-chamber view (A,D) and measurement of aortic annulus diameter (A).** The coronal and the short axis views of the aortic valve are shown (**B**, **C**).

When acquiring aortic valve using 3D technique, the probe was positioned in the mid esophageal position between 120° – 150°, and compression, gain and depth settings were optimized using a zoomed image. Real-time 3D imaging of a pyramidal volume of the aortic valve was obtained (average temporal resolution 20±3 frames/sec). All images were acquired during 3 cardiac cycles. The gathered 3D images of aortic valve were analyzed off-line (Q-LAB cardiac 3DQ, Philips, Andover, Massachusetts, USA). Using the multiplanar reformation (MPR) mode, standard short-axis views of the aortic valve were generated at the insertion of the cusps in systole. A sagittal view of the aortic annulus was obtained by placing a cut plane across a short-axis view centered on the ascending aorta, enabling the sagittal annular diameter to be recorded (Figure 2).

**Figure 3:**
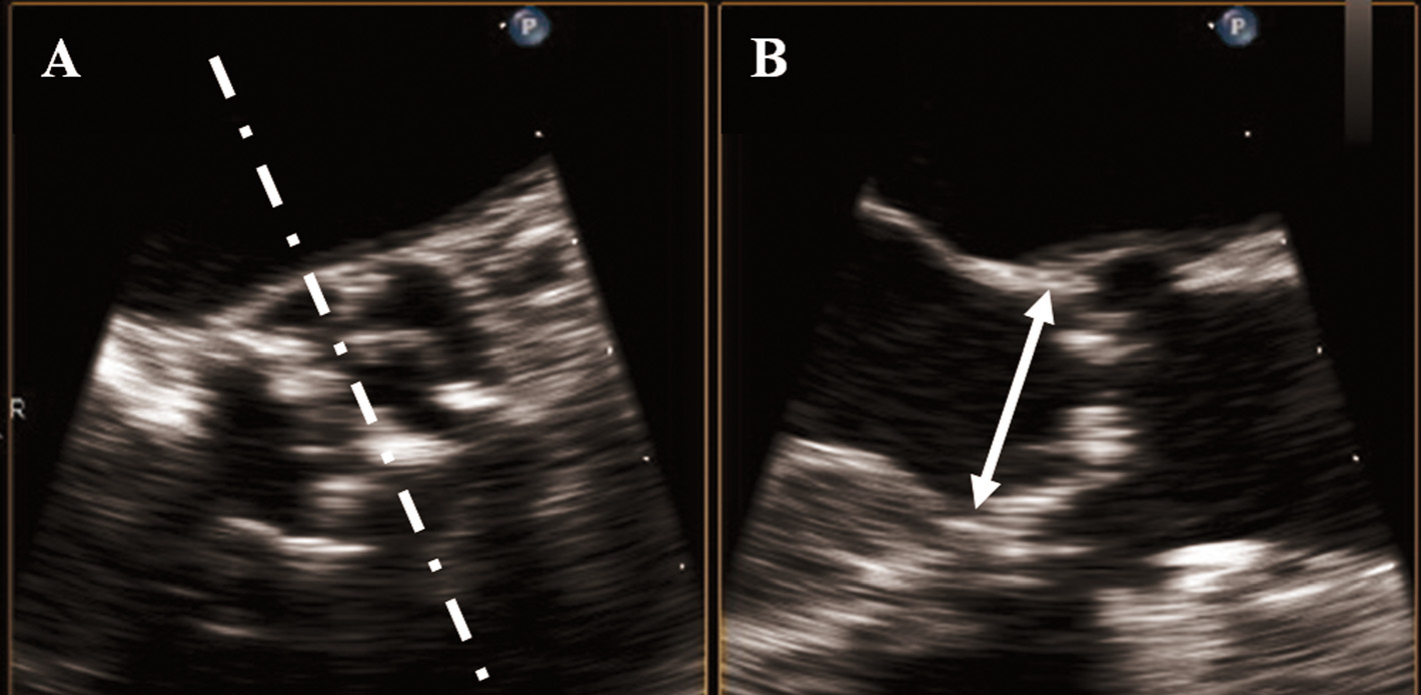
**Visualization of aortic valve by biplane-mode in short axis view (A) and the corresponding long axis view (B).** Every effort is made to place the cursor across the largest annulus diameter.

Imaging of the aortic valve in BP-mode was performed in a short axis view of the aorta. By positioning the X-plane across the aorta, the valve was visualized in long-axis (90° from short-axis view). Great care was taken to place the X-plane (cursor) in the midposition in the short-axis aortic valve view (Figure 3). Mean frame rate during acquisition of BP-mode images was 43±11 frames/sec.

### Statistical analysis

All statistical analyses were performed with IBM SPSS Statistics version 18.0 (IBM Corp., Armonk, NY, U.S.A.). Measurements of AoA obtained by different echocardiographic techniques were compared using Pearson´s correlation coefficient (r) and Bland-Altman analysis [23]. Comparison between variables was performed using analysis of variance (ANOVA). Suitability for TAVI (AoA > or < 20 mm) was tested between methods using McNemar’s test for proportions for paired data. In a subgroup of 20 patients, the measurements of AoA size were repeated by a separate, independent investigator in order to assess inter-observer variability. Observer variability was analyzed using the following formula: (SD_diff_ × 100%) / total mean × √2, where SD_diff_ is the SD of differences between measurements [24]. Statistical significance was considered present for p *<* 0.05. Continuous variables are expressed as mean ± SD.

## Results

No patients were excluded from the analysis due to poor image quality. The 3DTEE measurement of AoA dimension was the method used for the selection of prosthesis size.

Basic patient characteristics of the study population are shown in Table 1. Mean AoA dimension obtained by different imaging modalities is presented in Table 2. As shown, significantly smaller measurements were obtained by 2DTTE than with other imaging modalities (p < 0.0001). We found no statistically significant difference in AoA size between 2DTEE vs. BP-mode vs. RT3DE. However, limits of agreement between 2DTEE and other 3D imaging modalities for assessment of AoA dimension were relatively wide (2DTEE vs. BP-mode −0.6 ± 1.7 mm; 2DTEE vs. 3DTEE −0.8 ± 2.0 mm; Table 3). On the other hand, not only the correlation but also the agreement between BP-mode and 3DTEE for AoA size determination were high as illustrated in Bland-Altman analysis (mean difference −0.2 ± 0.9 mm, r = 0.88; Table 3).

**Table 1:** Basic patients characteristics (n=50)

**Variable**	**Value**
Age (years)	85±8
Male/female (n)	24/26
Body surface area (m^2^)	2.2±0.6
LV Ejection Fraction (%)	55±9
Heart rate (beats/min)	76±13
Peak jet velocity (m/s)	4.3±0.6
Mean gradient (mmHg)	50±16
Aortic valve area (cm^2^)	0.7±0.2
Index aortic valve area (cm^2^/m^2^)	0.4±0.1

LV, left ventricle.

**Table 2:** Aortic annulus measurements assessed by different echocardiographic modalities

**Modality**	**Aortic annulus diameter (mm)**
2DTTE	20.4±2.2*
2DTEE	22.3±2.5
BP-mode	22.9±1.9
3DTEE	23.1±1.9

*p < 0.0001 vs. 2D-TEE, BP-mode and RT3DE. 2D denotes two-dimensional; 3D, three-dimensional; TTE, transthoracic echocardiography; TEE, transesophageal echocardiography; BP, biplane.

**Table 3:** Agreement between different echocardiographic modalities

	**Mean difference ± SD**	**Limits of agreement**	**r-value**	p-value*
TTE-2DTEE	−2.0±2.1	−6.2 to 2.2	0.60	<0.05
TTE-BP	−2.5±1.9	−6.3 to 1.3	0.58	<0.05
TTE-3DTEE	−2.7±1.8	−6.3 to 0.9	0.64	<0.05
2DTEE-BP	−0.6±1.7	−4.0 to 2.8	0.72	<0.05
2DTEE-3D	−0.8±2.0	−4.8 to 3.2	0.62	<0.05
BP-3DTEE	−0.2±0.9	−2.0 to 1.6	0.88	<0.05

SD denotes standard deviation; TTE, transthoracic echocardiography; TEE, transesophageal echocardiography; BP, biplane; 3D, three-dimensional. *P-value for correlation coefficient.

There was evidence of misclassification the AoA in a subgroup of patients: the proportion regarded as having a too-small annulus to undergo TAVI was 2% with 3DTEE (n = 1). This figure was similar with BP-mode (4%, n = 2; p = 1.00) but considerably higher with both 2DTTE (36%, n = 18; p < 0.001) and 2DTEE (12%, n = 6; p = 0.06), indicating a large subgroup of patients were wrongly considered unsuitable for TAVI with 2DTTE.

### Variability

Interobserver variability was assessed in a subgroup of 20 patients. Interobserver reproducibility for the different imaging modalities were as follow: 3.2% for 3DTEE, 4.7% for 2DTTE, 4.0% for 2DTEE and 3.6% for BP-mode. Statistical analysis showed significant differences regarding reproducibility between 2DTTE and 2DTEE in comparison to BP-mode and 3DTEE (p<0.001).

## Discussion

A growing group of patients are offered AVR today in whom open heart surgery has previously been impossible due to co-morbidities and/or advanced age, following the advent of TAVI. It is crucial that correct measurements of AoA are obtained as implantation of a poorly matched prosthesis can have severe or even fatal consequences [7,25]. At present, there is no gold standard for non-invasive assessment of AoA dimension, and available techniques include different echocardiographic modalities as well as MSCT and angiography. However, TEE is typically frequently used as it enables the annulus to be directly visualized. In the current study, in a cohort of 50 patients referred for evaluation prior to TAVI, we found that taking AoA measurements by 2DTTE leads to significant underestimation and wide limits of agreement, as compared with other imaging modalities. While this is in agreement with previously published data in this field [12,26], it does challenge a recent report by Messika-Zeitoun *et al.* where no statistically significant difference was found between TTE and 2DTEE-based AoA measurements [13]. There are several possible explanations why AoA may be underestimated by 2DTTE. Apart from the aforementioned 2D orientation of the image plane, severe calcification and shadowing can make exact image interpretation and annulus measurements on TTE difficult. This limitation can be overcome using 3DTEE.

In patients undergoing TAVI with the Medtronic CoreValve System™, a 26 mm prosthesis is recommended for a 20 – 23 mm annulus and a 29 mm prosthesis in a 23 – 27 mm annulus [19]. TEE is recommended if the number of cusps cannot be determined [20]. The current recommendations for the use of echocardiography in transcatheter intervention suggest that 2DTEE and/or 3DTEE are used in patients whose 2DTTE-based AoA measurements are uncertain. This is especially pertinent when measurements are near critical cut-offs and in patients whose AoA cannot be determined due to calcification extending from aortic valve to either septum or anterior mitral leaflet. However, using TEE as a reference method has been associated with favorable clinical results [13,27], and TEE is accordingly the most widely used imaging modality for this purpose [6,28].

In our study, a measured AoA diameter < 20 mm was found in 36% (n=18) of the patient population using 2DTTE, but only in 12% (n=6) based on 2DTEE and 4% (n=2) and 2% (n=1) respectively, based on BP-mode and RT3DE images. This implies that a significant proportion of patients are in appropriately deemed to be unsuitable for TAVI when 2DTTE is used to measure AoA. Interestingly, in the conservatively managed arm of the PARTNER trial, mortality at 12 months was 51% with standard therapy compared to 31% with TAVI [29]. This demonstrates that the prognosis of patients that are wrongly declined TAVI is dismal and illustrates the potentially very large clinical implications of this finding.

Aortic annulus is described as a virtual ring formed by joining the basal attachments of the aortic cusps [9]. Several studies have shown that in many patients the aortic annulus is not circular but in fact oval in shape. This is likely to be the root cause behind the failure to measure AoA correctly in a single image plane [13,15,26,30,31], and explains the superiority of 3DE, which enables the larger coronal diameter of an ellipsoid AoA to be measured and not only the smaller sagittal.

We found good reproducibility in determination of AoA size with all echocardiographic modalities, the highest being with 3DTEE which has also been shown in other studies [12,31,32]. On the contrary, AoA measurements performed by CT has been demonstrated to have poor inter-observer variability [26]. While 3DTEE is widely used in this patient group, it requires post-processing at a dedicated workstation with appropriate software and training. Importantly, BP-mode and 3DTEE provided similar measurements (as evidenced by strong correlations) with similar inter-observer variability. Moreover, misclassification did not occur using BP-mode.

### Limitations

This study has several limitations. A major limitation is the lack of independent reference method like MSCT. The biplane mode technique for assessment of aortic annulus size is limited in terms of not to be able to measure the coronal diameter of the annulus and therefore the annulus area. This might be important since prosthesis/annulus area mismatch assessed by 3DTEE has recently shown to predict the outcome of significant paravalvular leak post TAVI [33]. Lastly, our study is limited by its lack of clinical outcome data.

## Conclusions

TAVI is offered to a very rapidly growing group of patients with severe aortic stenosis. We conclude that multi-plane imaging using BP-mode enables a speedy measurement to be taken of AoA which is both robust and exact. Moreover, a considerable proportion of patients are misclassified by single plane measurements as unsuitable for TAVI which is unfortunate and may have major clinical implications. This issue deserves to be studied in a prospective design in future research in this field.

## Competing interests

The authors declare that they have no competing interests.

## Authors’ contributions

KS initiated the study and included the patients and performed the image acquisitions together with MB. AS supervised the study and participated in the interpretation of the results and manuscript preparation. KS and AM performed measurements. KS and CdS performed statistical analysis of the manuscript and made all data conversions and plots. All authors read and approved the final manuscript.
